# Discretion: Whether and How Does It Promote Street-Level Bureaucrats' Taking Charge Behavior?

**DOI:** 10.3389/fpsyg.2022.805872

**Published:** 2022-05-24

**Authors:** Shuai Yuan, Zhixia Chen, Mei Sun

**Affiliations:** Department of Public Administration, College of Public Administration, Huazhong University of Science and Technology, Wuhan, China

**Keywords:** street-level bureaucrats, discretion, taking charge behavior, public service motivation, self-determination theory

## Abstract

The extant pieces of literature on discretion has mainly focused on its effect on policy implementation and public service delivery, but few studies have looked at its influence on street-level bureaucrats' work behavior, such as taking charge behavior (TCB), which is of great importance for government reforms, especially in developing and transitional countries. Based on the self-determination theory, this study examines whether and how discretion promotes street-level bureaucrats' TCB. Two studies were conducted among street-level bureaucrats in China. First, a survey experiment (*n* = 355) suggests that discretion positively predicts street-level bureaucrats' TCB. Then, a survey questionnaire study (*n* = 442) shows that discretion is positively related to TCB through the mediator of public service motivation (PSM). We concluded with implications for theory and practice.

## Introduction

Taking charge behavior (TCB) refers to a type of voluntary and constructive behavior of individual employees to promote organizationally functional change within the contexts of their jobs, work units, or organizations (Morrison and Phelps, 1999), which is also called change-oriented organizational citizenship behavior (Choi, 2007; Kim et al., 2011; Love and Dustin, 2014; Chen D. C. et al., 2021). In recent years, with the organizational environment showing high competitiveness and uncertainty, TCB has become critical to individual performance and organizational effectiveness (Fuller and Marler, 2009; Kim et al., 2015). Researchers have thus devoted attention to the TCB of employees in the private sector (Moon et al., 2008; Burnett et al., 2015; Li et al., 2016; Zhou et al., 2020), but the TCB of public sector employees, particularly street-level bureaucrats, has received little attention. However, significant processes of reforms have taken place in governments over the last few decades (Fattore et al., 2017), and many public sector, particularly in developing and transitional countries such as China, are now under pressure to make changes (Homberg et al., 2019). As a result, public sectors are increasingly encouraging employees, especially street-level bureaucrats, to engage in TCB because of its potential to benefit its reforms (Homberg et al., 2019). Specifically, street-level bureaucrats' TCB is necessary and essential not only for the implementation of top-down reforms and the achievement of intended reform objectives, since most reforms are executed by street-level bureaucrats (Ahmad et al., 2019, 2020; Hassan et al., 2020), but also for bottom-up policy innovation, as they sometimes become policy entrepreneurs (Durose, 2007; Arnold, 2015). However, TCB is a kind of discretionary change-oriented behavior (Seppälä et al., 2012). There are many significant risks for street-level bureaucrats in conducting TCB (Morrison and Phelps, 1999; Parker et al., 2010). First, people tend to resist changes (Frese and Fay, 2001). In addition, TCB can create conflict and harm relationships (McAllister et al., 2007), which may reduce street-level bureaucrats' willingness to perform TCB (Li et al., 2016). This may be particularly true in the context of Chinese culture emphasizing on collectivism (Chen Z. X. et al., 2002) and harmonious relationships between leaders and coworkers (Tjosvold et al., 2006). Therefore, finding measures to promote street-level bureaucrats' TCB has become crucial.

Street-level bureaucrats are the frontline workers of public administration; they act as both clientele agents and state agents in the implementation of policies and the delivery of public services (Gassner and Gofen, 2018). Their discretion is one of the defining features that sets them apart from other public sector employees. Although scholars have discussed and affirmed the importance and necessity of discretion (Hupe and Hill, 2007; Pires, 2010; Tummers and Bekkers, 2014), previous research has mainly focused on its important role in policy implementation and public service delivery (Thomann et al., 2018; however, the influence of discretion on street-level bureaucrats' work behaviors is hitherto overlooked. In part, this study fills the gap in the literature by expanding on the research on the motivational effect of discretion on street-level bureaucrats' work behaviors, especially on their TCB.

Taking charge behavior has potential risks because of its challenging nature (Morrison and Phelps, 1999; McAllister et al., 2007). Parker et al. (2010) identified “reason to” motivation and “can do” motivation as leading factors that motivate individuals to conduct proactive behavior, including TCB. Furthermore, based on self-determination theory (SDT), Parker et al. (2010) and Li et al. (2016) elaborated on how autonomous motivation could drive an individual's TCB. Therefore, the main purpose of this study is to investigate and analyze whether discretion could predict street-level bureaucrats' TCB, building on the line of SDT. We argue that discretion contributes to the construction of street-level bureaucrats' “reason to do” motivation and “can do” motivation, which then stimulates their TCB, because discretion allows them to make decisions autonomously and helps them feel efficacious in tackling public issues.

Meanwhile, as a kind of intrinsic motivation (Ward, 2014), PSM, a general altruistic intrinsic motivation to serve the interests of citizens, nations, or humankind (Rainey and Steinbauer, 1999), has been reported to have a direct and positive relationship with street-level bureaucrats' TCB (Homberg et al., 2019). Besides, PSM can be characterized as autonomous motivation, and it also relates to satisfaction with the basic needs (Jensen and Bro, 2018). The needs for autonomy and competence are arguably important for intrinsic motivation and PSM (Corduneanu et al., 2020). Moreover, although previous research generally assumes PSM as a stationary and unchangeable inherent motive (Perry and Wise, 1990), recent research suggests that PSM can be changeable (Vandenabeele, 2014; Ward, 2014; Han, 2018; Chen C. A. et al., 2021). Discretion helps street-level bureaucrats perceive themselves as the initiators of their actions and as competent individuals in benefiting clients; thus, we argue that discretion may stimulate street-level bureaucrats' PSM and that discretion further has an indirect effect on street-level bureaucrats' TCB *via* PSM. These assumptions have previously not been subjected to empirical investigation. Based on this notion, this article attempts to explore whether and how discretion can stimulate street-level bureaucrats' TCB by examining the mediating role of PSM.

In so doing, our research makes several contributions. First, the study investigated the direct effect of discretion on street-level bureaucrats' TCB following SDT, which will provide a theoretical understanding of the positive outcomes of discretion. Second, our research contributed to the literature regarding TCB of public sector employees, hence expanding the occupational categories for TCB. Third, we also contributed to the literature on TCB by expanding its antecedents. This research explored the relationship between discretion and TCB and found another antecedent of TCB. Fourth, our study contributed to the literature on discretion and TCB by examining the mediating role of PSM, which helps to open the “black box” of the mediation mechanism from discretion and TCB. Fifth, we conducted both a survey experiment and a survey questionnaire to test our hypotheses successively, which is conducive to assure the internal and external validities of the study.

In the next section, we will review the literature and introduce our hypotheses based on the previous research. Then, two studies using different methods are conducted to test the hypotheses: a survey experiment establishes variations in the scope of discretion to explore whether discretion will predict street-level bureaucrats' TCB, and a survey questionnaire is conducted to explore the effect mechanism between discretion and TCB. Finally, after presenting the results, we conclude and discuss how our results can inform public administration scholars and practitioners.

## Literature Review And Hypotheses

### Discretion and Street-Level Bureaucrats' TCB

TCB is, by definition, self-initiated and autonomous (Parker et al., 2010); it encourages employees to focus on the future of the organization, discover the procedures and structures that restrict organizational efficiency, its changes (Homberg et al., 2019), and contribute to its innovation (Morrison and Phelps, 1999). Street-level bureaucrats' TCB is conducive to efficiently correcting and perfecting work procedures and patterns (Dan and Vicki, 2006) and promoting reforms in the public sector (Homberg et al., 2019). Specifically, street-level bureaucrats' TCB includes introducing and adopting improved approaches for better public service, eliminating redundant or unnecessary procedures to improve efficiency, and even changing rules or policies that are non-productive or counterproductive.

Because TCB brings about constructive changes and often poses challenges to the organizational status quo (Homberg et al., 2019), motivation and competence are essential components of street-level bureaucrats' TCB due to its challenging nature (Parker and Collins, 2010; Cai et al., 2018). Based on previous research and SDT, we assume that discretion is expected to drive street-level bureaucrats' TCB because it contributes positively to facilitating their intrinsic autonomous motivation and self-efficacy to engage in TCB. The rationale for this hypothesis is as follows:

First, employees who can accomplish their work autonomously are more likely to conduct TCB (Homberg et al., 2019; Chen D. C. et al., 2021), and discretion contributes to the satisfaction of street-level bureaucrats' basic psychological needs, including the need for autonomy. As the necessary tool for street-level bureaucrats to perform duties (Jones, 2001), discretion allows them to make decisions in specific situations (Thomann et al., 2018), as well as to choose between possible courses of action and inaction (Hupe and Hill, 2007). Therefore, discretion not only compensates for the policy defects (Canales, 2011) but also boosts the willingness of street-level bureaucrats to implement policies (Tummers and Bekkers, 2014). Relying on discretion, street-level bureaucrats decide how to deliver public services (Wenger and Wilkins, 2009; Morten et al., 2019), decide which clients can be helped, and how to help them (Belabas and Gerrits, 2017). Consequently, street-level bureaucrats act autonomously in meeting clients' needs and improving clients' welfare and wellbeing, which contributes to constructing their “reason to do” motivation for TCB. Following SDT (Deci and Ryan, 2000), we propose that the satisfaction of basic psychological needs for autonomy will improve street-level bureaucrats' autonomous motivation to engage in TCB. Besides, according to Fuller et al. (2006), employees feel a more personal responsibility for their own work products as job autonomy increases, which means that discretion may help street-level bureaucrats feel responsibility for constructive change, making them more likely to engage in TCB.

Second, “can do” motivation is also an important determinant of TCB because of the potential risk to the individual (Parker et al., 2010); that is, street-level bureaucrats tend to assess the likelihood of success and possible consequences before engaging in TCB (Morrison and Phelps, 1999). Discretion offers the opportunities to experiment, which greatly improves street-level bureaucrats' self-efficacy. Street-level bureaucrats with a high level of self-efficacy may show a higher level of TCB (Chen D. C. et al., 2021). Besides, the opportunities to experiment could also increase the likelihood of street-level bureaucrats successfully carrying out TCB and reduce the potential risks of consequences. In sum, the following hypothesis is proposed:

H1: Discretion positively predicts street-level bureaucrats' TCB.

### Mediating Effect of PSM

Following previous research and SDT, we further inferred that discretion may have an influence on TCB through PSM. PSM, as noted, is a type of autonomous and intrinsic motivation (Ward, 2014); it relates to the satisfaction of the basic psychological needs for autonomy and competence (Jensen and Bro, 2018). Moreover, PSM is an important factor in explaining public sector employees' behaviors, especially proactive behavior (Homberg et al., 2019). Administrators with a higher PSM are often willing to bring benefits to others and to the society by providing better public service (Perry et al., 2008); hence, they would be inclined to promote public benefits through their jobs (Lewis and Frank, 2002; Vandenabeele, 2008) and they are more likely to engage in constructive organizational behavior; thus, recruiting employees with a higher PSM will be more likely to contribute to public sector reforms and public service delivery in both quantity and quality (Cerase and Farinella, 2009). Therefore, in our study, we propose that PSM plays a mediating role through which the influence of discretion on street-level bureaucrats' autonomous and intrinsic motivation for TCB is channeled.

Discretion helps street-level bureaucrats believe that they are autonomous and capable administrators; it contributes to satisfy their needs for autonomy and competence and further facilitates their PSM. In most instances, street-level bureaucrats have made efforts to meet clients' needs and improve clients' welfare and wellbeing (Evans, 2013); however, they often work under the conditions of scarce governance resources, heavy workloads, and conflicting demands (Sommer, 2018) and work in situations that require responses to citizens' multidimensional needs (Lipsky, 2010). Their desire for discretion reflects their need for autonomy in their work (Lammers et al., 2016). Discretion allows street-level bureaucrats to tailor policies to specific circumstances (Thomann et al., 2018) so that they can act on their own decisions when responding to the needs of clients (Tummers and Bekkers, 2014). Then, discretion endows street-level bureaucrats with autonomy in dealing with complex affairs, allowing them to bend the rules (Canales, 2011), which improves their self-efficacy for policy implementation and makes them play an important role in policy implementation (Barnes and Julia, 2018), as well as helps them feel that they can make a difference in clients' lives (Maynard-Moody and Musheno, 2003), thereby increasing their enthusiasm and initiative to serve clients. In short, discretion gets street-level bureaucrats to believe that they can autonomously and effectively deal with specific public affairs and respond to citizens' requirements, which contributes to satisfying their basic psychological needs for autonomy and competence. Following SDT (Gagné and Deci, 2005), their intrinsic motivation and PSM will be improved (Jensen and Bro, 2018; Corduneanu et al., 2020).

Street-level bureaucrats with a higher PSM tend to be more concerned with the benefits of the organization and its clients (Rainey and Steinbauer, 1999) and hence, they are more likely to exhibit altruistic behavior and proactive behavior. Empirical evidence suggested that PSM is positively related to street-level bureaucrats' TCB (Homberg et al., 2019). At the same time, there are also empirical evidence that suggests that administrators with a higher PSM are less likely to resist changes (Wright et al., 2013a; Hassan et al., 2020), are more likely to accept and support organizational change (Naff and Crum, 1999; Cerase and Farinella, 2009; Hassan et al., 2020), and maintain a supportive attitude toward government reform efforts because they perceived those reforms to benefit the organization and its citizens (Wright et al., 2013a). In addition, PSM is found to have a positive effect on employees' change-oriented organizational citizenship (Campbell and Im, 2016) and innovative behaviors (Miao et al., 2017). That is, street-level bureaucrats with a higher PSM would be more inclined to conduct TCB to support and contribute to organizational changes because of their willingness to benefit the organization and its clients (Wright et al., 2013a). They prefer to adopt new approaches because they align with their personal goals and values to make timely and effective responses to citizens' public service requirements.

Taken together, we assume that discretion reinforces PSM, which, in turn, provokes TCB. In other words, when street-level bureaucrats enjoy discretion, their PSM will be enhanced because of the satisfaction of their basic psychological needs for autonomy and competence (Jensen and Bro, 2018; Corduneanu et al., 2020) and they will be more likely to perform TCB to benefit the organization and its clients driven by altruistic motivation (Rainey and Steinbauer, 1999). Therefore, discretion will motivate street-level bureaucrats to conduct TCB, al least partly on account of discretion which contributes to the enhancement of PSM. Thus, the hypothesis is proposed:

H2: PSM mediates the relationship between discretion and TCB.

**Figure 1:**
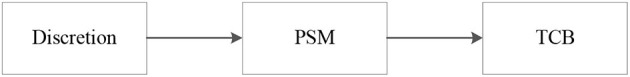
Conceptual model.

The conceptual model is depicted in Figure 1.

## Empirical Studies

### Study 1: Survey Experiment on the Discretion and TCB

#### Research Design

We conducted a survey experiment to test the effect of discretion on TCB. The survey experiment has become a popular methodology across the social sciences (Mullinix et al., 2015). It uses hypothetical scenarios to reproduce the work environment so that researchers can control institutional settings (James et al., 2017), which allows us to manipulate the scope of street-level bureaucrats' discretion.

The survey was conducted from December 2020 to February 2021 in China. The study used a single-factor simple inter-group design. Two vignette scenarios were developed: much discretion and little discretion. Alternate randomization was used to ensure the statistical comparability of the subjects between different groups. We printed two kinds of questionnaires, one for the much discretion group and the other for the little discretion group, and numbered the questionnaires successively (from 1 to 400) according to the above order. The serial numbers of the much discretion group were odd numbers, and the serial numbers of the little discretion group were even numbers. Each group was provided with 200 questionnaires. The numbered questionnaires were distributed successively to ten interviewers, and then every interviewer distributed the questionnaires to subjects successively *via* WeChat (see Qin et al., 2018, 2020, for studies that used WeChat for data collection) or *via* a paper-and-pencil form.

We sent out questionnaires to 400 front-line civil servants in China, including police officers, community workers, social workers, Chengguan officers (urban management and law enforcement officers in China), traffic police, market supervisors, and government service centers clerks. Although they come from different public sector occupations, they all deal directly with citizens in their work, and importantly, all of them enjoy some degree of discretion in their work.

Participants were told to participate in an experiment about administrators' work behaviors. All participants read a vignette scenario describing a work situation and then completed the questionnaire. As shown in Table 1, participants in the much discretion condition read a vignette scenario with much discretion. Conversely, participants in the little discretion group read a vignette scenario with little discretion.

**Table 1:** Vignette scenarios.

**Participants**	**Vignette scenario**
Much discretion group	Imagine that you are a front-line civil servant who deals with citizens directly, such as traffic police, police officers, market supervisors, and so forth. In your work, under the premise of abiding by laws and regulations, you are allowed to make decisions freely about the procedure, time limits, and methods of handling affairs. You have the freedom to decide which working methods and procedures should be adopted when you think there are more rational and efficient working methods and procedures. When some special circumstances occur, you have flexibility in dealing with them.
Little discretion group	Imagine that you are a front-line civil servant who deals with citizens directly, such as traffic police, police officers, market supervisors, and so forth. In your work, laws and regulations have made clear instructions for the procedure, time limits, and methods of handling affairs. You must strictly abide by the relevant regulations. Even if you find that there are more rational and efficient working methods and procedures, you have no right to adapt these methods or procedures.

Next, all participants completed the TCB items. TCB was measured using 10 items developed by Morrison and Phelps (1999). The responses were recorded using a 5-point Likert scale (1 = strongly disagree, 5 strongly agree). The sample item is “I try to adopt improved procedures.” The alpha reliability of this measure was 0.864. Then, all participants completed one manipulation check item, assessing the extent to which our manipulations of vignette scenarios affected their perceptions of discretion, where the item read: “In your work, you can make some flexible treatments according to the actual conditions within the legal framework.” Participants responded on a scale of 1 (strongly disagree) to 7 (strongly agree).

We received 372 questionnaires. After removing the incomplete questionnaires, we finally obtained 355 valid samples (88.75%), with 178 valid samples from the much discretion group and 177 valid samples from the little discretion group.

#### Analyses

Table 2 shows the observable respondents' characteristics and the group difference tests on gender, age, education, and tenure. The results confirm that the randomization standard between the groups was satisfied.

**Table 2:** Valid sample characteristics (means and standard deviations in parentheses, *N* = 355).

**Variables**	**Much discretion**	**Little discretion**	**Groups difference test**
Gender	1.45 (0.50)	1.52 (0.50)	Chi-square = 1.76, *p* = 0.19
Age	2.03 (0.52)	2.04 (0.53)	ANOVA, *F* = 0.01, *p* = 0.92
Education	2.75 (0.52)	2.81 (0.57)	ANOVA, *F* = 1.01, *p* = 0.30
Tenure	2.88 (1.05)	2.71 (1.11)	ANOVA, *F* = 2.36, *p* = 0.13
*N*	178	177	

#### Results

We assessed whether scenarios influence participants' responses to the manipulation check item. To test the effectiveness of the manipulation, an independent sample *t*-test shows that participants in the much discretion group (*M* = 4.46, *SD* = 1.57) argued that they had more discretion than participants in the little discretion group (*M* = 3.58, *SD* = 1.74), *t*_(353)_ = 4.99, *p* < 0.001, Cohen's *d* = 0.53 (Cohen, 1992). The results show that the manipulation was effective.

Then, we tested whether discretion predicts street-level bureaucrats' TCB. In line with hypothesis 1, an independent samples *t*-test shows that participants in the much discretion group (*M* = 3.66, *SD* = 0.55) are likely to perform more TCB than participants in the little discretion group (*M* = 3.25, *SD* = 0.56), *t*_(353)_ = 6.75, *p* < 0.001, *Cohen's d* = 0.72 (Cohen, 1992).

The results favor our hypothesis 1, that is, when street-level bureaucrats have more discretion and are allowed to make decisions about what should be done and how to make these decisions, they would be willing to perform more TCB. However, Study 1 fails to explain how discretion promotes street-level bureaucrats' TCB; therefore, Study 2 will explore the facilitating mechanism between them.

### Study 2: How Does Discretion Arouse TCB

In line with previous research, Study 2 intends to analyze the mediating role of PSM between discretion and TCB.

#### Methods and Samples

Study 2 used a survey questionnaire, which was conducted from March 2021 to May 2021 in China. We administered 500 questionnaires to front-line civil servants from different public sectors simultaneously *via* WeChat and *via* a paper-and-pencil form. Similarly, they all interact directly with citizens and exercise discretion in their work. We received 442 valid samples (88.4%) from 462 participants after removing incomplete questionnaires. Table 3 shows the characteristics of valid samples.

**Table 3:** Frequency counts for categorical variables (*N* = 442).

**Variables**	**Variable levels**	**Count**
Gender	Women	174
	Men	268
Age	~25	46
	26~35	337
	36~45	47
	46~55	10
	Over 56	2
Education	Master	122
	Bachelor	285
	Associate	35
Tenure	0~1	44
	1~3	148
	4~6	119
	7~10	83
	Over 10	48

#### Measures

We adapted the discretion (autonomy) scales with three items developed by Hackman and Oldham (1980) to measure the scope of discretion. The sample item was “I have significant autonomy in determining procedures of my job.” The alpha reliability of this measure was 0.711. Public service motivation was measured using a 5-item scale developed by Wright et al. (2013b). The sample item was “Meaningful public service is very important to me.” The alpha reliability of this measure was 0.741. TBC was measured using a ten-item scale by Morrison and Phelps (1999). The sample item was “I often try to adopt improved procedures.” The alpha reliability of this measure was 0.906.

Control variables: Following the previous research on TCB (Fuller et al., 2012; Li et al., 2016), this study considered participants' gender, age, education level, and tenure as control variables.

All responses were recorded using a 5-point Likert scale (1 = strongly disagree, 5 = strongly agree).

## Results

### Descriptive Statistics and Correlations

The results of descriptive statistics and correlations of variables are displayed in Table 4. The results suggest that there are significant and positive relationships between discretion, PSM, and TCB, which give initial support to our hypotheses.

**Table 4:** Descriptive statistics.

**Variables**	***M***	**SD**	**1**	**2**	**3**	**4**	**5**	**6**
1. Gender	1.610	0.489						
2. Age	2.060	0.582	−0.035					
3. Education	2.800	0.563	−0.019	0.201^***^				
4. Tenure	2.870	1.158	−0.070	0.530^***^	0.201^**^			
5. Discretion	3.086	0.922	−0.042^**^	0.154^**^	0.136^**^	0.115^*^		
6. PSM	3.880	0.650	−0.008^*^	0.131^**^	0.051	0.061	0.361^***^	
7. TCB	3.479	0.707	−0.111^**^	0.169^***^	0.064	0.109^*^	0.555^***^	0.467^***^

*PSM, Public service motivation; TCB, Taking charge behavior*.

* *p < 0.05*,

** *p < 0.01*,

*** *p < 0.001*.

### Common Method Bias, Reliability, and Validity

We adapted ULMC to test the common method bias (Podsakoff et al., 2003). Table 5 suggests that the difference between the model with the proposed factors (3-factor model: D, PSM, TCB) and the model loaded with the common source latent factor (4-factor model: CMV, D, PSM, TCB) was insignificant (ΔRMR = 0.017, ΔGFI = 0.015, ΔIFI = 0.017, ΔTLI = 0.013, ΔCFI = 0.016, ΔRMSEA = 0.006), which revealed that the common method bias might not be a serious problem in our study.

**Table 5:** Results for confirmatory factor analysis.

**Models**	**CMIN/DF**	**RMR**	**GFI**	**IFI**	**TLI**	**CFI**	**RMSEA**
Four-factor model (CMV; D; PSM; TCB)	2.101	0.031	0.947	0.967	0.952	0.966	0.050
Hypothesized three-factor model (D; PSM; TCB)	2.402	0.048	0.932	0.950	0.939	0.950	0.056
Two-factor model (D and PSM are combined into)	4.507	0.070	0.855	0.873	0.847	0.872	0.089
Single-factor model	5.807	0.074	0.821	0.825	0.791	0.824	0.104

*D, Discretion; PSM, Public service motivation; TCB, Taking charge behavior; CMV, Common method variance*.

Then, we used Amos 24.0 to conduct a confirmatory factor analysis for the scale; the results are shown in Table 5. The results show that the three-factor model has the best model fit indexes than any other alternative model; in other words, the discretion, PSM, and TCB involved in this study could be distinguished from each other, and they were distinct and representative of the constructs.

### Test of Hypotheses

We applied multiple OLS regression analyses to test our hypotheses; the results are shown in Table 6. We firstly assess the hypothesized direct relationship (H1). H1 assumes a positive relation between discretion and TCB and is fully supported, the coefficient on discretion (β = 0.542, *p* < 0.001) is positive and highly significant in model 4, hence we conclude that discretion is a driver of TCB.

**Table 6:** Results of multiple regression analysis (robust standard errors in parentheses).

**Variables**	**PSM**		**TCB**			
	**M 1**	**M 2**	**M 3**	**M 4**	**M 5**	**M 6**
**Controls**
Gender	−0.004 (0.063)	0.008 (0.059)	−0.104^*^ (0.068)	−0.086^*^ (0.057)	−0.102^*^ (0.061)	−0.088^*^ (0.054)
Age	0.134^*^ (0.063)	0.093 (0.059)	0.151^**^ (0.068)	0.089 (0.057)	0.091 (0.061)	0.061 (0.054)
Education	0.027 (0.056)	−0.011 (0.053)	0.028 (0.061)	−0.030 (0.051)	0.016 (0.054)	−0.026 (0.048)
Tenure	−0.016 (0.032)	−0.026 (0.030)	0.016 (0.034)	−0.001 (0.029)	0.023 (0.030)	0.007 (0.027)
**Hypotheses**
Discretion		0.351^***^ (0.032)		0.542^***^ (0.031)		0.436^***^ (0.031)
PSM					0.452^***^ (0.046)	0.302^***^ (0.043)
*R^2^*	0.018	0.137	0.041	0.324	0.241	0.402
*ΔR^2^*	0.018	0.119^***^	0.041^***^	0.283^***^	0.201^***^	0.161^***^
*F*	1.988	13.789^***^	4.617^**^	41.659^***^	27.741^***^	48.765^***^

* *p < 0.05*,

** *p < 0.01*,

*** *p < 0.001*.

Then, we investigated the mediation hypothesis (H2) using the Baron and Kenny (1986) approach. Model 2 tests the H2 by regarding PSM as a dependent variable to explore the relationship between the discretion and potential mediator, PSM. Model 5 displays the association of PSM with TCB, and Model 6 is the full model. The results suggest that discretion significantly affects PSM (β = 0.351, *p* < 0.001), and PSM is a significant predictor of TCB (β = 0.452, *p* < 0.001). Meanwhile, Model 6 provides evidence for partial mediation as the association between discretion and TCB decreases but remains significant (β = 0.436, *p* < 0.001). Therefore, we conclude that the H2 gets supported, establishing PSM as a mediator in the relationship between discretion and TCB.

## Discussion

Based on the self-determination theory and precious research, we proposed a theoretical model of street-level bureaucrats' discretion and their TCB, which provides a preliminary picture of whether and how discretion influences TCB. We conducted two studies using the survey experiment and the survey questionnaire. The model and all the hypotheses are fully supported by the empirical data from street-level bureaucrats in China. Specifically, we found that discretion positively predicts street-level bureaucrats' TCB, and PSM plays a partially mediating role in the relationship.

### Theoretical Contributions

The study makes several theoretical contributions. First, the most important contribution is that we have constructed and tested a theoretical model that focuses on the influence of discretion on street-level bureaucrats' TCB based on SDT. Previous research on discretion primarily concentrated on its effects on policy implementation and public service delivery (Tummers and Bekkers, 2014; Thomann et al., 2018). Nevertheless, as the necessary tool for street-level bureaucrats to perform duties (Jones, 2001), discretion inevitably has an important impact on their work behaviors. The positive relationship between discretion and TCB found in this study suggests that discretion can promote street-level bureaucrats' TCB by increasing their intrinsic motivation. The conclusion broadens the scope of street-level bureaucrats' work behaviors motivated by discretion, which also enriches the positive outcomes of discretion.

Second, this study has enriched the research on the TCB of street-level bureaucrats. As mentioned above, some pieces of research discussed the TCB of employees in the private sector (Moon et al., 2008; Burnett et al., 2015; Li et al., 2016; Zhou et al., 2020), yet the TCB of public sector employees, especially street-level bureaucrats, has been ignored. However, the pressure for changes and reforms in public sectors have created a demand for street-level bureaucrats to perform TCB (Ahmad et al., 2019, 2020; Homberg et al., 2019). To fill this gap, our study provided a concrete demonstration of the importance of discretion in developing street-level bureaucrats' TCB.

Third, our study expanded the antecedents of street-level bureaucrats' TCB by clarifying the influence of discretion on TCB. Many researches have proved that TCB was positively impacted by individual factors, such as knowledge/abilities (Fay and Frese, 2001) and personal initiative (Frese and Fay, 2001), as well as situational factors, such as leadership styles (López-Domínguez et al., 2013; Li et al., 2016; Bilal et al., 2019) and supportive organizational climate (Griffin et al., 2007). Our study focused on street-level bureaucrats and found the important role of another factor—discretion—in promoting TCB.

Fourth, our study proposed that PSM has a mediating effect on the relationship between discretion to TCB. This study found that discretion boosts TCB by facilitating street-level bureaucrats' PSM. The autonomous motivation stimulated by discretion can be positively transmitted through PSM. The finding explicated an important theoretical model to illustrate why street-level bureaucrats with much discretion will be more likely to conduct TCB, which helps to open the “black box” of the mediation mechanism from discretion to TCB.

Fifth, we conducted both a survey experiment and a survey questionnaire to test our hypotheses. The survey questionnaire is one of the prevalent research methods of prior studies on public employees' organizational behavior. However, this approach is criticized for its limited internal validity. Our research tested the hypotheses using a survey experiment and a survey questionnaire successively, which was conducive to assure the internal and external validities of the study.

### Practical Implications

Our study also makes several practical contributions. First, this study suggests that discretion contributes to the satisfaction of the basic psychological needs for autonomy and competence and further motivates street-level bureaucrats to engage in TCB, which is important because public sector reforms rely heavily on employees' reactions to change (Ahmad et al., 2020). Therefore, public sectors that want to encourage street-level bureaucrats' TCB should value the significance of discretion and empower street-level bureaucrats with a certain amount of discretion. Although there are risks of abuse of discretion, increasing street-level bureaucrats' discretion may be more conducive to policy implementation than curbing it in some conditions (Lipsky, 2010), which also benefits TCB. However, to avoid and reduce the risks associated with the abuse of discretion, proper training should be required (Cárdenas and Ramírez de la Cruz, 2017; Morten et al., 2019).

Second, considering the positive mediating effect of PSM between discretion and TCB, as well as the fact that street-level bureaucrats with a high PSM will show strong self-supervision of their work behaviors (Weber et al., 2004), we suggest that public managers should select street-level bureaucrats with a high PSM (Ahmad et al., 2019). Alternatively, given recent research indicating that PSM is changeable (Vandenabeele, 2014; Ward, 2014; Han, 2018; Chen C. A. et al., 2021), public managers can also conduct training programs to cultivate street-level bureaucrats' PSM.

### Limitations and Future Directions

Although we make efforts to perfect the study, some limitations remain. First, discretion does sometimes result in administrative evil. There is no doubt that discretion is a double-edged sword, and when we emphasize its importance, we should maintain vigilance against the abuse of discretion. Without proper controls, training, and knowledge, discretion will result in an efficient but unfair condition (Cárdenas and Ramírez de la Cruz, 2017). We suggest that public managers should empower street-level bureaucrats with discretion and have control over the process of exercising discretion fairly (Shim et al., 2020); however, we are unable to give a conclusion on how much discretion should be granted to street-level bureaucrats to empower them. Future research should analyze the appropriate level of discretion according to the overall country context, the level of governance, type of task environment, and other factors.

Second, our study has not considered organizational factors that likely impact street-level bureaucrats' TCB. Evidently, organizational factors have important and continuous effects on street-level bureaucrats' TCB, such as perceived organizational support, which is positively associated with TCB (Li et al., 2016; Homberg et al., 2019). However, we remind readers that our study warrants only an illustration of the relationship between discretion and TCB. Future studies should focus on the effect of organizational factors on street-level bureaucrats' TCB.

Third, the study used subjective survey data obtained from a single source of street-level bureaucrats. Although CMV was not a major issue in our study, future studies should present a longitudinal study to confirm the relationship between discretion and TCB.

## Data Availability Statement

The original contributions presented in the study are included in the article/supplementary material, further inquiries can be directed to the corresponding author.

## Author Contributions

SY designed the study, performed the data analysis, and wrote the draft of the manuscript. ZC revised the manuscript. MS evaluated and revised the manuscript. All authors contributed to the article and approved the submitted version.

## Funding

This work was supported by Key Project of National Social Science Foundation of China (Nos. 20AZD019 and 17AGL014), Chinese Science Foundation of Ministry of Education (18JHQ080), and Huazhong University of Science and Technology Humanities and Social Sciences Double First-Class Construction Project Fund (HUST 2019).

## Conflict of Interest

The authors declare that the research was conducted in the absence of any commercial or financial relationships that could be construed as a potential conflict of interest.

## Publisher's Note

All claims expressed in this article are solely those of the authors and do not necessarily represent those of their affiliated organizations, or those of the publisher, the editors and the reviewers. Any product that may be evaluated in this article, or claim that may be made by its manufacturer, is not guaranteed or endorsed by the publisher.
